# Systemic T Cell Subsets and Cytokines in Patients With Homozygous Sickle Cell Disease and Asymptomatic Urinary Tract Infections in Togo

**DOI:** 10.31486/toj.20.0061

**Published:** 2021

**Authors:** Christèle Nguepou Tchopba, Gnatoulma Katawa, Essohana Padaro, Pélagie Edlom Tchadié, Simplice Damintoti Karou, Ahoefa Vovor, Yaovi Ameyapoh

**Affiliations:** ^1^ Unité de Recherche en Immunologie et Immuno-modulation (UR2IM)/Laboratoire de Microbiologie et de Contrôle Qualité des Denrées Alimentaires (LAMICODA)/Ecole Supérieure des Techniques Biologiques et Alimentaires (ESTBA)-Université de Lomé, Togo, West Africa; ^2^ Département des Sciences Fondamentales, Faculté des Sciences de la Santé (FSS)-Université de Lomé, Togo, West Africa

**Keywords:** *Asymptomatic urinary tract infections*, *cytokines*, *immune response*, *sickle cell disease*, *T helper cells*, *Togo*

## Abstract

**Background:** In sickle cell disease (SCD), cytokine expression influences the pivotal pathways that contribute to disease pathogenesis. Additional infection could affect the immune profile of patients with SCD and increase disease mortality. The aim of this study was to investigate the cytokines and T helper cells profile in patients with asymptomatic urinary tract infection and homozygous SCD (HbSS).

**Methods:** From July to September 2018, 22 HbSS subjects were recruited at Centre Hospitalier Universitaire Campus in Lomé, Togo, 12 of whom had urinary tract bacterial infections and 10 of whom were uninfected. Cytokines from plasma were measured by the enzyme-linked immunosorbent assay (ELISA) sandwich method, and immune cell profiles were performed by flow cytometry. The immunogenicity of bacteria-derived antigens isolated from the urine of HbSS subjects with asymptomatic urinary tract infections was studied in a cell culture system, and the induction of the cytokines was measured.

**Results:** The mean age of HbSS subjects with urinary tract infections was 20.33 ± 3.58 years, and the male/female ratio was 0.09 (1:11). HbSS subjects with asymptomatic urinary tract infections had elevated plasma levels of interferon gamma (IFN-γ) and interleukin (IL)-10. CD4^+^Tbet^+^IFN-γ^+^ and CD4^+^FoxP3^+^IL-10^+^ T cell populations were decreased in HbSS subjects with asymptomatic urinary tract infections. Bacterial antigens from HbSS subjects induced the production of IL-10 but not IFN-γ in uninfected volunteer donors (HbAA).

**Conclusion:** Our study demonstrated that patients with SCD and asymptomatic urinary tract infections had elevated IFN-γ and IL-10 levels. This chronic inflammatory condition could be a risk for this group of patients in terms of vaso-occlusive crisis. Systematic cytobacteriologic examination of the urine of HbSS subjects would be of interest.

## INTRODUCTION

Sickle cell disease (SCD) is a neglected genetic disease, especially in Africa, where an estimated 50% to 90% of patients die undiagnosed before their fifth birthdays.^1^ Globally, approximately 300,000 infants per year are born with homozygous SCD (HbSS).^2^ In Togo, a country of the West African region with approximately 7.889 million people, SCD is the most common hemoglobin (Hb) abnormality.^3^ The frequency of the HbS gene has been estimated at 16.1%, the prevalence of HbSS at 1.3%, and the prevalence of the double heterozygous forms (HbSC and HbS/β-thalassemia [βthal]) at 2.6%. Approximately 200,000 Togolese are estimated to have a major form of SCD (HbSS, HbSC, or HbS/βthal).^3^

Patients with SCD have an increased inflammatory profile compared to healthy individuals (HbAA). The chronic inflammatory state in SCD is associated with factors such as endothelial damage; increased production of reactive oxygen species; hemolysis; increased expression of adhesion molecules by leukocytes, erythrocytes, and platelets; and increased production of proinflammatory cytokines.^4^ In SCD, cytokine expression influences the pivotal pathways that contribute to disease pathogenesis.^5^ Some authors have noted that the level of inflammatory cytokines is higher during a vaso-occlusive crisis than in steady state.^6-8^

Infections significantly contribute to morbidity and mortality in patients with SCD.^9,10^ Bacterial infection, one of the principal complications of SCD, can occur as an acute or chronic condition.^9,11^ Patients with SCD have an increased risk of urinary tract infection (UTI), and kidney diseases are usually more severe in HbSS patients compared to HbAA individuals^12^; kidney diseases account for 16% to 18% of mortality in patients with SCD.^13^

Asymptomatic UTI is defined as the isolation of a specific number of bacteria (more than 10^3^ to 10^5^ bacteria/mL depending on the species) from a urine sample that was collected in an appropriate manner, but the patient does not have symptoms or signs related to a UTI.^14^ Such a situation can be a prelude to a high risk of pyelonephritis, kidney failure, or even sepsis.^15^

Immune response during a UTI is highly activated. Innate immunity acts through the mechanical action of eliminating urine and also activates inflammation to eliminate bacteria. Adaptative immunity is involved for bacterial elimination through T helper or CD4^+^ T lymphocytes.^16^ Inflammatory, anti-inflammatory, and regulatory cytokines, which are mediators of the immune response, are also involved.^17^

In 2019, we found that approximately 9.3% of Togolese patients with homozygous SCD had an asymptomatic UTI.^18^ Because SCD is an inflammatory disease and knowing that UTIs activate inflammation, we hypothesized that UTIs may exacerbate the inflammatory immune response in patients with SCD.

For this study, we investigated the systemic cytokines and T helper cells profiles in Togolese patients with homozygous SCD and asymptomatic UTIs.

## METHODS

### Study Population and Sample Collection

Subjects enrolled for this study have been previously described.^18^ The study population was recruited at Centre Hospitalier Universitaire Campus (CHU Campus) in Lomé, Togo, which had a clinical hematology unit for the follow-up of patients with SCD living in Togo.

From July to September 2018, 129 patients with SCD were consecutively recruited for cytobacteriologic examination of urine. Included in this study were patients with the HbSS genotype who were followed at the CHU Campus; were in steady state according to medical opinion; did not take antibiotics during the prior 2 weeks^19^; were not taking any medications; and did not have kidney disease, diabetes, or any other disease. We found that 12 patients had UTI bacteria at a significant level. From the recruited patients with SCD, we selected 10 HbSS subjects free of UTI bacteria. For comparison, we recruited heterozygous blood-related HbAS subjects (n=10) and healthy volunteers (HbAA) (n=11) who were members of the CHU Campus laboratory staff. HbAS and HbAA subjects were free of UTI bacteria. Blood samples were collected at the CHU Campus in Lomé, Togo, and immunological assays were performed at Unité de Recherche en Immunologie et Immunomodulation of Laboratoire de Microbiologie et de Contrôle des Denrées Alimentaires of Ecole Supérieure des Techniques Biologiques et Alimentaires-Université de Lomé.

### Cytobacteriologic Examination of Urine

Cytobacteriologic examination of urine was performed for all subjects. A sterile vial and instructions were given for the collection and transport of urine samples. Participants collected their urine samples at home early in the morning (approximately 6:00 am) at least 4 hours after the last urination and in strict hygienic conditions. The samples were received from 7:00 am to 9:30 am at the bacteriology laboratory on the CHU Campus. Samples were stored at 4 °C from time of receipt until the start of handling. All urine samples were cultured, using a sterile calibrated loop, on Uri*Select* 4 chromogenic medium (Bio-Rad Laboratories, Inc) for 18 to 24 hours at 37 °C. The remaining volume of urine was then centrifuged at 4,000 rpm for 5 minutes. The pellets were examined for leukocytes, red blood cells, urinary crystals, granular cylinders, and parasites. Leukocyturia was determined on a Nageotte cell and was considered significant if 10^4^ cells/mL. Bacterial identification was performed, and bacteriuria was determined by counting bacterial colonies on an agar plate and multiplying the result by the loop volume (×10).^18^

### T Helper Cell Profiling

**Figure 1.**
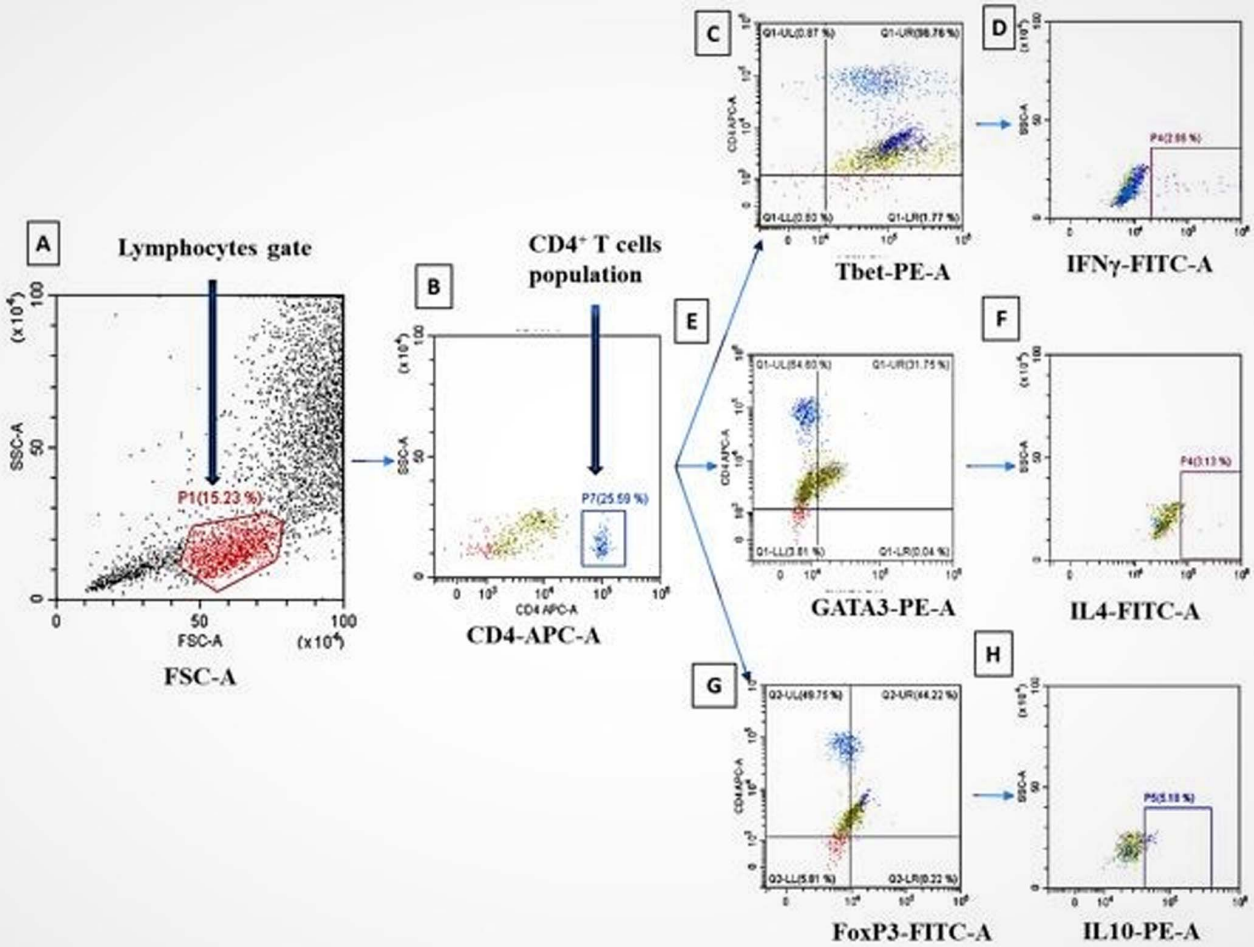
**Gating strategy: (A) Lymphocyte gate; (B) CD4^+^ T lymphocyte population (LTCD4^+^) delineated among the lymphocytes; (C) (E) (G) are respectively CD4^+^Tbet^+^, CD4^+^GATA3^+^, and CD4^+^FoxP3^+^ dot plots; (D) CD4^+^Tbet^+^IFN-γ^+^ T cells are IFN**-**γ^+^ cells delineated among CD4^+^Tbet^+^ cells; (F) CD4^+^GATA3^+^IL-4^+^ T cells are IL-4^+^ cells delineated among CD4^+^GATA3^+^ cells; and (H) CD4^+^FoxP3^+^IL-10^+^ T cells are IL-10^+^ cells delineated among CD4^+^FoxP3^+^ cells.**

To assess the T helper cell profile of the study population, 100 μL of whole blood cells was activated in duplicate with 50 μL of 1× concentrated cell stimulation cocktail plus protein transport inhibitors containing 40 μM of phorbol 12-myristate 13-acetate (PMA), 670 μM of ionomycin (as antibiotic), 5.3 mM of Brefeldin A (as protein transport inhibitor), and 1 mM of Monensin, all in 500× concentrated ethanol (Invitrogen/Thermo Fisher Scientific) for 6 hours at 37 °C under 5% carbon dioxide (CO_2_). Samples were collected, and red blood cells were lysed with 200 μL of red blood cell lysis buffer (Roche Diagnostics) according to manufacturer instructions. Cells were then stained for T helper type 1 (Th1), T helper type 2 (Th2), and natural regulatory T (nTreg) cells surface markers; intracellular cytokines; and transcription factors. Surface staining was performed using antihuman CD4-APC antibody (clone A16A1) and incubated at 4 °C for 30 minutes. Then, cells were washed and permeabilized using Fix & Perm reagent (Invitrogen/Thermo Fisher Scientific). After blocking Fc receptors with TruStain FcX (BioLegend), cells were stained in 3 different panels: (1) Th1 as antihuman interferon gamma (IFN-γ)-FITC (clone 4S.B3) and Tbet-PE (clone 4B10); (2) Th2 as antihuman interleukin (IL)-4-FITC (clone MP4-25D2) and GATA3-PE (clone 16E10A23); and (3) nTreg as antihuman IL-10-PE (clone JES3-9D7) and antihuman FoxP3-FITC (clone 206D). After 15 minutes at 4 °C, cells were acquired on CytoFLEX flow cytometer (Beckman Coulter), and data were analyzed with CytExpert 2.1 software (Beckman Coulter). Fluorescence compensation was done using VersaComp Antibody Capture Bead Kit (Beckman Coulter) to correct spectral overlap. The gating strategy is depicted in Figure 1.

### Bacterial Antigen Preparation

We investigated the immunogenicity of 3 clinical strains of *Escherichia coli* (randomly named S1, S2, and S3) isolated from the urine of HbSS subjects. These bacterial strains were obtained after 24-hour urine culture on Uri*Select* 4 agar and identified. Thereafter, bacterial strains were stored on microbeads (Microorganism Preservation System – Protect, Technical Service Consultants Ltd) at –20 °C for 3 months. Bacteria were then cultured on the Uri*Select* 4 medium for 24 hours at 37 °C. One colony was selected and cultured in Oxoid Mueller-Hinton Broth (ThermoFisher Scientific). Bacteria were inactivated at 56 ± 1 °C for 6 hours in a water bath,^20^ followed by triple washing with Dulbecco's phosphate buffered saline (DPBS) (ThermoFisher Scientific). To assess the complete inactivation, a bacterial culture on brain heart infusion agar (Liofilchem, Inc) was performed.

After complete inactivation of bacteria, tubes containing the culture broth were centrifuged, and the pellet was collected and added to 1 mL of DPBS. The protein concentration was measured by Bradford assay. Bacterial antigens obtained were frozen at –20 °C for further use.

### Isolation of Peripheral Blood Mononuclear Cells

Blood samples (20 mL) in K3 EDTA tubes from 6 healthy volunteer donors from the national blood bank of Lomé were collected, and peripheral blood mononuclear cells (PBMCs) were isolated by the Ficoll density gradient centrifugation method described by Katawa et al.^21^ In brief, 20 mL of whole blood was diluted in 15 mL of DPBS and carefully added to 15 mL of Pancoll separating solution (PAN-Biotech). After 20 minutes’ centrifugation at 2,000 rpm, the white layer of PBMCs was collected and washed 3 times in Roswell Park Memorial Institute (RPMI) medium supplemented with gentamicin 50 μg/mL, penicillin-streptomycin 100 μg/mL, L-glutamine 2 mM/mL, and fetal bovine serum (FBS) at 10% (PAN-Biotech). Cells were counted, and their viability was assessed using the trypan blue exclusion method.

### In Vitro Cell Culture

For the cell culture performed in a 96-well U-bottom plate (Greiner Bio-One), 2 × 10^5^ PBMCs per well were incubated, in duplicate, without or with 1 μg/mL of bacteria extracts and with 1 ng/mL of lipopolysaccharide (InvivoGen) as positive control^22^ in RPMI medium supplemented with FBS at 10%. Cells were then incubated at 37 °C under 5% CO_2_ for 24 hours and 72 hours.

### Cytokines Measurement

Blood samples were spun at 4,000 rpm for 5 minutes, and plasma was collected. Plasma samples of subjects and culture supernatants were stored at –20 °C until experimental analysis. Samples were thawed once for type 1 (IL-6 and tumor necrosis factor [TNF]-α) and type 2 (IFN-γ, IL-5, and IL-10) cytokine measurement by the sandwich enzyme-linked immunosorbent assay (ELISA) method using an Invitrogen kit (ThermoFisher Scientific). Cytokine concentrations were measured using a HumaReader HS (HUMAN Diagnostics Worldwide).

### Statistical Analysis

Sample size was calculated according to a 2010 study from Nigeria, in which the researchers found that 4% of patients with SCD have asymptomatic bacteriuria.^23^ Using the formula *n*=[*Z*1/2^2^ * *p*(1-*p*)]/*d*^2^, where *n* is the sample size, *p* is the prevalence of the disease in the population, *Z*1/2^2^ is equal to 1.96, and *d* is the tolerable sampling error (0.05),^23^ we found that the minimal sample size required for our study was 59.

Statistical analyses were performed using Prism, version 5.02 (GraphPad Software Inc). To compare cytokines and cell profiles, a Mann-Whitney *U* test was used to compare 2 groups. The mean ages of the 4 study groups were compared using a Kruskal-Wallis test. Chi-square test was used to compare the ratio of ages (<18/≥18 years) and the ratio of sexes. *P* values <0.05 were considered significant.

### Ethics Approval and Consent to Participate

This study was approved by the Bioethics Committee for Health Research from the Togo Ministry of Health (No. 15/2018/CBRS). Written informed consent was obtained from all subjects. For minors, a parent's consent was obtained.

## RESULTS

### Characteristics of the Studied Populations

Demographic characteristics of the studied populations are shown in the Table. We found no significant difference in the mean ages of the 4 groups, but we found significant differences in the age ratio (<18/≥18 years) and the sex ratio among the 4 groups (*P*=0.011, *P*=0.018, respectively).

**Table.** Demographic Characteristics of the Studied Populations

	Population				
Variable	HbSS With Infection^a^ n=12	HbSS^b^ n=10	HbAS^c^ n=10	HbAA^d^ n=11	*P* Value
Age, years, mean ± SD	20.33 ± 3.58	21.10 ± 2.31	26.00 ± 2.89	27.27 ± 1.70	0.106
Age ratio: <18 years:≥18 years	6:6	5:5	1:9	0:11	0.011
Sex ratio: male:female	1:11	4:6	5:5	8:3	0.018

^a^HbSS with infection, subjects with homozygous sickle cell disease and with urinary tract infection.

^b^HbSS, subjects with homozygous sickle cell disease and without urinary tract infection.

^c^HbAS, heterozygous subjects without urinary tract infection.

^d^HbAA, subjects without sickle cell disease or sickle cell trait and without urinary tract infection.

The mean age of HbSS subjects who had a UTI was 20.33 ± 3.58 years, and the ratio of males to females was 1:11. The uninfected subjects (HbSS, HbAS, or HbAA) had leukocyturia <10^4^ cells/mL and no significant bacteriuria (<10^5^ bacteria/mL), whereas the infected subjects had leukocyturia ≥10^4^ cells/mL and bacteriuria: ≥10^3^ bacteria/mL for *E. coli* (for males and females); ≥10^3^ bacteria/mL for *Staphylococcus* spp (for males); ≥10^4^ bacteria/mL for *Staphylococcus* spp (for females); ≥10^5^ bacteria/mL for *Enterococcus* spp, *Streptococcus* spp, and *Enterobacter* spp (for males and females). In cases of leukocyturia <10^4^ cells/mL, bacteriuria ≥10^5^ bacteria/mL was considered UTI positive for all bacteria.

The most common strains of bacteria responsible for the UTIs were *E coli* (66.67%), *Enterobacter* spp (8.33%), *Staphylococcus* spp (8.33%), *Enterococcus* spp (8.33%), and *Streptococcus* spp (8.33%).

### Elevated Plasma Levels of IFN-γ and IL-10 in Infected HbSS Subjects

**Figure 2.**
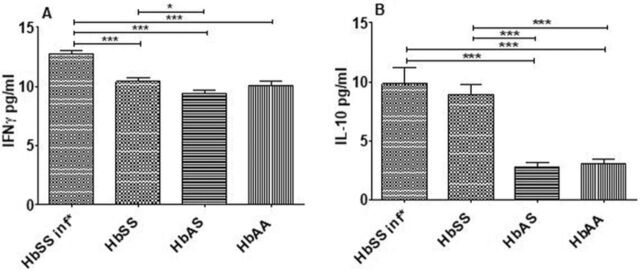
Plasma cytokine profile measured by sandwich enzyme-linked immunosorbent assay method. The graphs show plasma levels of (A) interferon gamma (IFN-γ) and (B) interleukin-10 (IL-10) in HbSS subjects who had urinary tract infections (HbSS inf*, n=12), HbSS subjects who did not have urinary tract infections (HbSS, n=10), uninfected HbAS subjects (n=10), and uninfected HbAA volunteers (n=11). Bars indicate the concentration of cytokines in each group. Data are expressed as mean ± SEM. Asterisks show statistical differences (Mann-Whitney *U* test) between the groups (*P<0.05).

HbSS subjects presenting with UTI had elevated plasma levels of IFN-γ and IL-10 compared to uninfected HbSS, HbAS, and HbAA subjects (Figure 2). Levels of IFN-γ were high in uninfected HbSS subjects compared to HbAS subjects. No differences were found between subjects with HbAS and HbAA. IL-10 was also high in uninfected HbSS subjects compared to HbAS and HbAA subjects. IL-5, IL-6, and TNF-α were not detected in the plasma of all groups.

**Figure 3.**
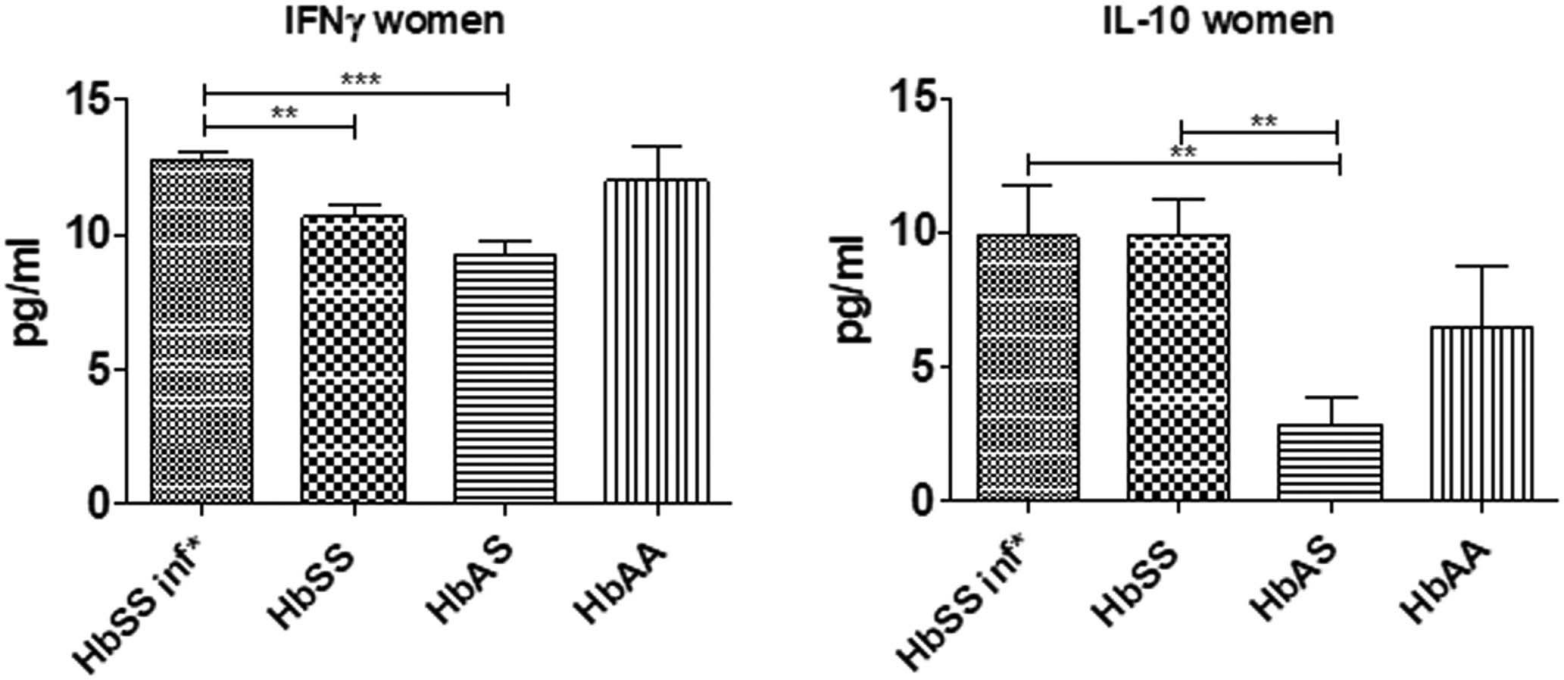
Plasma cytokine profile in females only measured by sandwich enzyme-linked immunosorbent assay method. The graphs show plasma levels of (left) interferon gamma (IFN-γ) and (right) interleukin-10 (IL-10) in HbSS subjects who had urinary tract infections (HbSS inf*, n=11), HbSS subjects who did not have urinary tract infections (HbSS, n=6), uninfected HbAS subjects (n=5), and uninfected HbAA volunteers (n=3). Bars indicate the concentration of cytokines in each group. Data are expressed as mean ± SEM. Asterisks show statistical differences (Mann-Whitney *U* test) between the groups (*P<0.05).

We compared the cytokine profile in females only (Figure 3). HbSS females with UTI secreted more IFN-γ than uninfected HbSS and HbAS females, and the difference was significant. A significant difference was also seen in IL-10 levels; HbSS females, both infected and uninfected, secreted more IL-10 in their plasma than uninfected HbAS females.

### Profile of CD4^+^ T Cells

**Figure 4.**
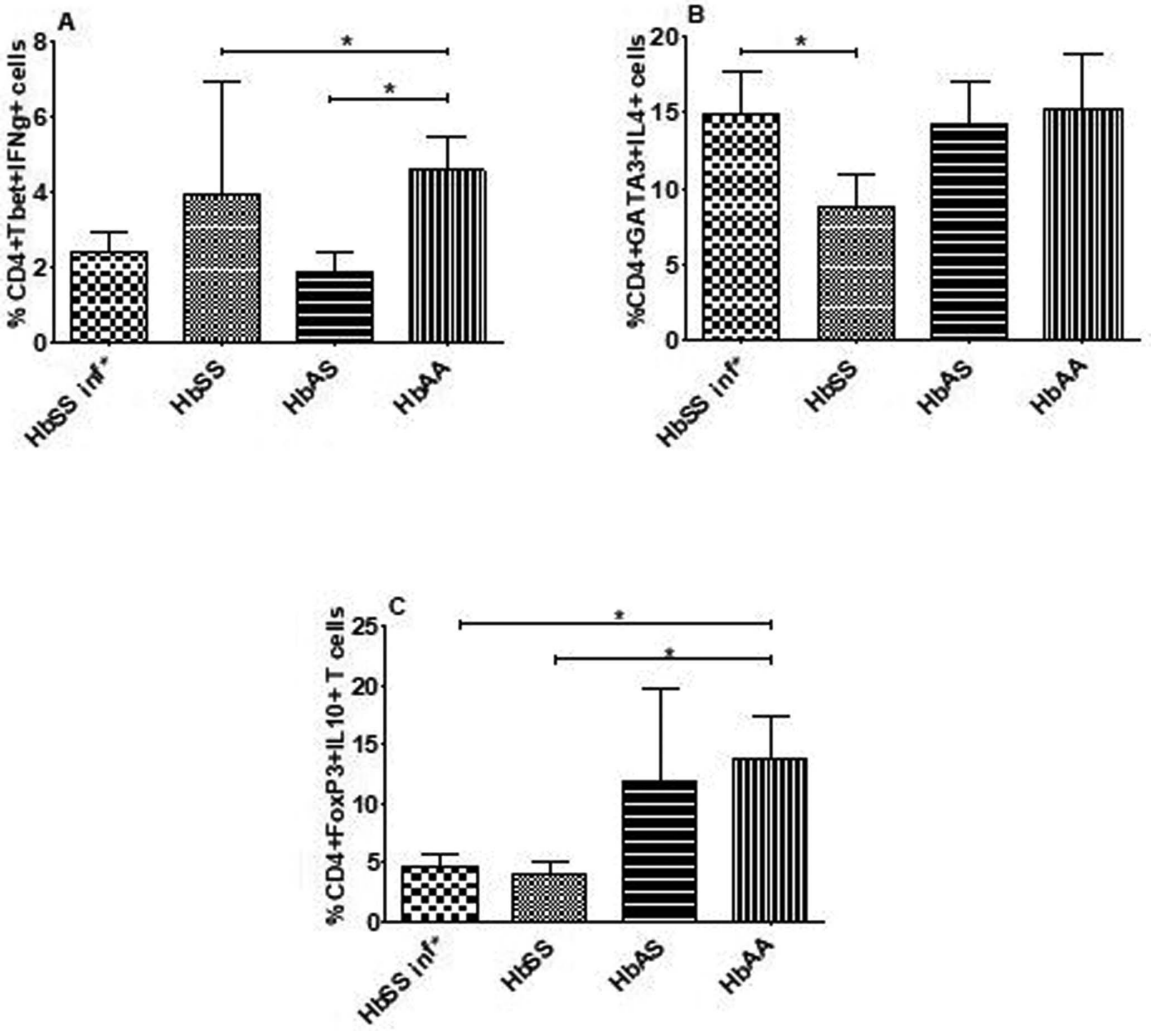
T helper type 1, T helper type 1, and natural regulatory T cells profile. Cells from HbSS subjects who had urinary tract infections (HbSS inf*, n=12), HbSS subjects who did not have a urinary tract infection (HbSS, n=10), uninfected HbAS subjects (n=10), and uninfected HbAA volunteers (n=11) were activated with phorbol 12-myristate 13-acetate and stained to determine the frequencies of (A) CD4^+^Tbet^+^IFN-γ^+^ T cells, (B) CD4^+^GATA3^+^IL-4^+^ T cells, and (C) CD4^+^FoxP3^+^IL-10^+^ T cells. Bars indicate the percentages of cells, expressed as mean ± SEM. Asterisks show statistical differences (Mann-Whitney *U* test) between the groups (*P<0.05).

To investigate the source of cytokines of the study population, CD4^+^ T cells were characterized (Figure 4). CD4^+^Tbet^+^IFN-γ^+^ and CD4^+^FoxP3^+^IL-10^+^ T cell populations were decreased in HbSS subjects with asymptomatic UTI***.*** However, the percentage of CD4^+^Tbet^+^IFN-γ^+^ T cells was significantly higher in uninfected HbSS subjects compared to uninfected HbAA subjects. Further, the percentage of CD4^+^Tbet^+^IFN-γ^+^ T cells was significantly lower in uninfected HbAS subjects vs uninfected HbAA subjects.

The percentage of CD4^+^GATA3^+^IL-4^+^ T cells in infected HbSS subjects was significantly higher than the percentage in uninfected HbSS subjects.

The percentages of CD4^+^FoxP3^+^IL-10^+^ T cells in infected and uninfected HbSS subjects were significantly lower than the percentage in uninfected HbAA subjects.

These results indicate that the primary source of IFN-γ and IL-10 in the plasma of infected HbSS subjects was not from Th1 or nTreg cells.

### Antigens From Strains Isolated From HbSS Subjects With Asymptomatic Bacteriuria Induced TNF-α, IL-6, and IL-10

**Figure 5.**
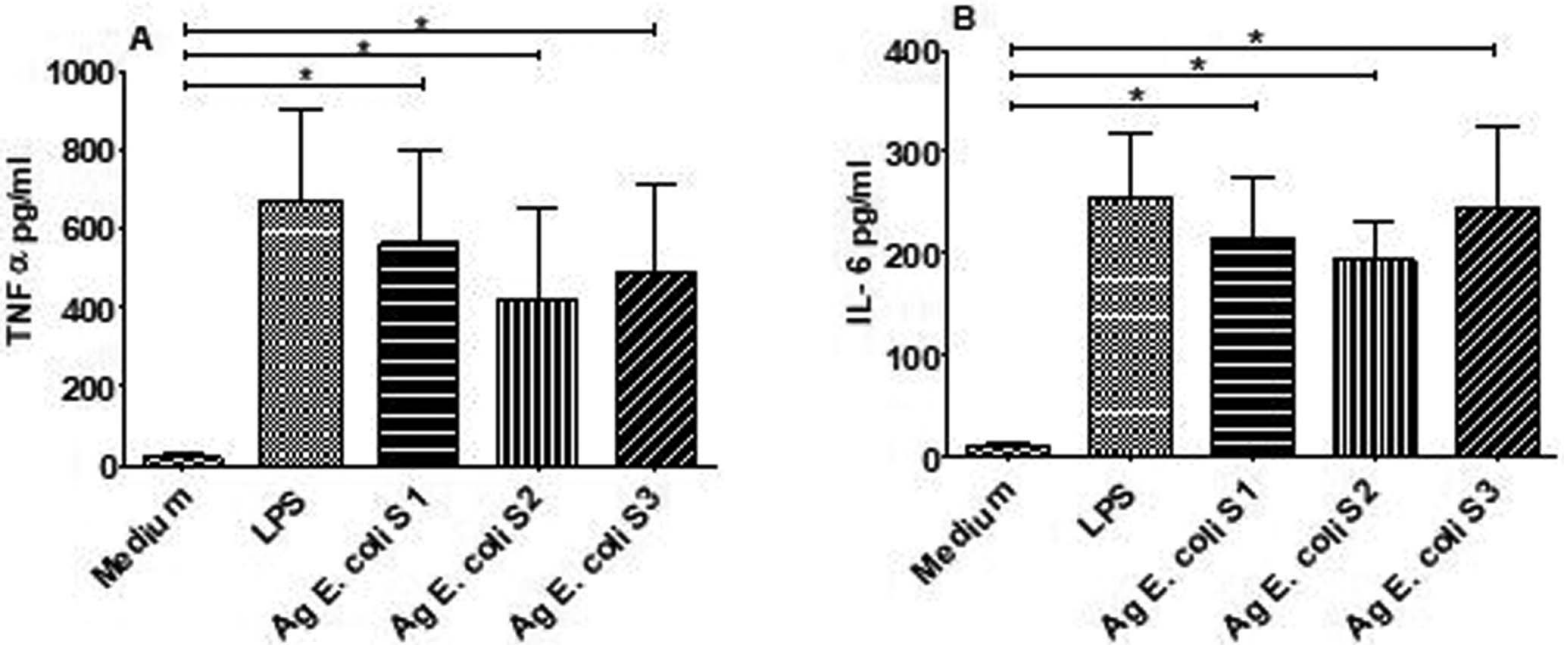
Immunogenicity of bacterial strains isolated from the urine of infected HbSS subjects after 24 hours of culture. Peripheral blood mononuclear cells (PBMCs) from healthy volunteer donors (n=6) were stimulated with antigens (Ag) derived from bacterial strains (randomly named S1, S2, and S3). Bars indicate levels of type 1 cytokines: (A) tumor necrosis factor (TNF)-α and (B) interleukin (IL)-6. Medium indicates the well containing unstimulated cells. LPS indicates the well containing cells co-cultured with lipopolysaccharide. Asterisks show statistical differences (Mann-Whitney *U* test) between the different co-cultures or PBMC stimulations performed (*P<0.05).

We examined the capacity of antigens from clinical bacterial strains isolated from the urine of HbSS subjects to induce inflammatory responses. PBMCs from HbAA healthy donors were stimulated with antigens from 3 strains of *E coli* derived from asymptomatic infected HbSS subjects. All 3 bacterial strains induced significant production of proinflammatory cytokines TNF-α and IL-6 after 24 hours of culture compared to the unstimulated cells (Figure 5).

**Figure 6.**
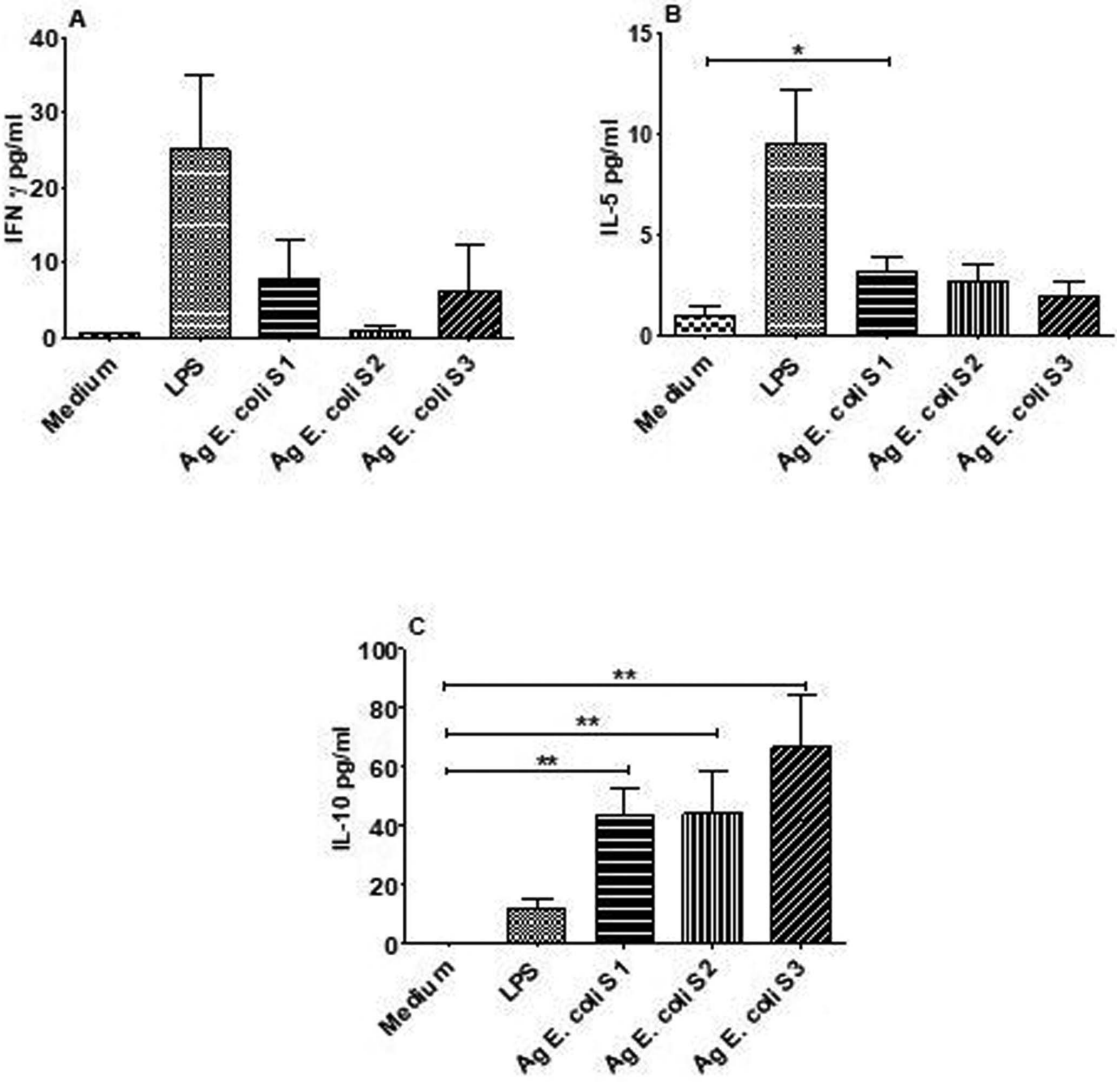
Immunogenicity of bacterial strains isolated from the urine of infected HbSS subjects after 72 hours of culture. Peripheral blood mononuclear cells (PBMCs) from healthy volunteer donors (n=6) were stimulated with antigen (Ag) from bacterial strains (S1, S2, and S3). Bars indicate levels of type 2 cytokines: (A) interferon gamma (IFN-γ), (B) interleukin (IL)-5, and (C) IL-10. Medium indicates the well containing unstimulated cells. LPS indicates the well containing cells co-cultured with lipopolysaccharide. Asterisks show statistical differences (Mann-Whitney *U* test) between the different co-cultures or PBMC stimulations performed (*P<0.05).

However, bacterial strains isolated from asymptomatic infected HbSS subjects did not induce significant production of IFN-γ after 72 hours of culture (Figure 6A). Only *E coli* S1 induced more IL-5 production compared to the unstimulated cells (Figure 6B). In contrast to the IFN-γ results, all *E coli* strains isolated from infected HbSS subjects induced elevated IL-10 production compared to the unstimulated control (Figure 6C).

## DISCUSSION

We investigated the systemic cytokine and T helper cell profiles of asymptomatic UTI in HbSS subjects in Togo. We observed that infected HbSS subjects had a significantly higher level of IFN-γ than uninfected HbSS subjects. We also found that HbSS subjects, both infected and uninfected, had significantly higher plasma levels of IL-10 than HbAA and HbAS subjects (Figure 2). In addition, we could not detect IL-5, IL-6, or TNF-α in the plasma of any study group. Investigating if Th cells were the main source of cytokines, we realized that the cytoplasmic and plasmatic cytokine levels did not follow the same trend.

*E coli* antigens from infected HbSS subjects co-cultured with PBMCs of healthy donors induced significant production of TNF-α and IL-6 (Figure 5). Furthermore, all *E coli* antigens responsible for asymptomatic UTI induced significant production of IL-10 (Figure 6).

The pathophysiology of SCD is driven by polymerization of HbS, leading to hemolysis and vaso-occlusive events; inflammation is a fundamental and constant component in these processes and can lead to tissue damage.^24^ Among bacterial infections in humans, UTI is prevalent,^25^ and patients with SCD have an increased susceptibility to bacterial infection.^26^ UTIs are more prevalent in females than males,^27^ and females were the majority of our infected HbSS study population, with a male to female ratio of 1:11. The implication of Th cells in UTI remain unclear.^28^

Infected HbSS subjects had a significantly higher rate of IFN-γ than uninfected HbSS subjects. IFN-γ is recognized as a key player in cellular immunity; it can orchestrate many protective functions to enhance immune responses in infections.^29^ Jones-Carson et al demonstrated that IFN-γ is protective against UTIs; they found that IFN-γ-knockout C57BL/6 mice compared to wild type mice were more likely to develop a UTI^30^; however, an acute/chronic inflammatory state can be damaging to tissues and organs.^31^

The cytokine profile data observed for females only (Figure 3) were approximately the same as the data observed in the 2 sexes (Figure 2), except we found no significant difference between the profiles of HbSS females and HbAA females. The lack of difference could be explained by the sample sizes: 3 HbAA females vs 11 infected HbSS females and 6 uninfected HbSS females.

The plasma level of IL-10 in infected and uninfected HbSS subjects was higher than that in uninfected HbAA and HbAS subjects, and the difference was significant (Figure 2). As an immunomodulatory cytokine, IL-10 plays a central role in limiting the host immune response to pathogens, preventing host damage and maintaining normal tissue homeostasis.^32^ IL-10 is elevated during the steady state of SCD compared to normal controls.^8,23^ Because patients with SCD in this study were in steady state, the level of IL-10 is justified.

In patients with UTIs, IL-10 may protect the host against exaggerated immune responses that produce inflammation and tissue damage.^16,33^ An argument can therefore be made that high IL-10 levels are protective and may explain the absence of common UTI symptoms such as fever, pelvic pain, and micturition pain in infected patients.

TNF-α, IL-6, and IL-5 were undetectable in the plasma of all study groups. TNF-α and IL-6 are more implicated than other cytokines in severe UTIs.^17^ Siransy et al revealed high levels of IL-6 in patients with SCD in crisis.^34^ IFN-γ, elevated as in this study, has been found to inhibit IL-5 production.^35^ Because our study groups consisted of asymptomatic UTI-infected HbSS subjects in steady state and uninfected HbSS, HbAS, and HbAA subjects, the levels of TNF-α, IL-5, and IL-6 may well be undetectable.

Several immune cells could release cytokines measured in plasma.^36^ We found that the primary sources of IFN-γ and IL-10 in the plasma of infected HbSS subjects were neither Th1 nor nTreg cells. Because the Th lymphocytes are not the source of the cytokines, we think that IFN-γ and IL-10 are produced by macrophages. The 3 types of macrophages are naïve macrophages, classically activated macrophages that secrete IFN-γ, and alternatively activated macrophages that secrete IL-10.^37^ In cases of bacterial aggression, the bacteria stimulate the pathogen recognition receptors of immune cells—macrophages in this case—through pathogen-associated molecular patterns or damage-associated molecular patterns, and an immune response follows. For example, lipopolysaccharides, a major component of the *E coli* outer membrane, binds to the toll-like receptor 4 (TLR4) of the macrophage, resulting in the production of IFN-γ by the macrophage through a signaling pathway to interferon regulatory factor (IRF)-3/7 and nuclear factor-κB (NF-κB) involving the TRAM and TRIF adaptors.^38^ In addition, *E coli* lipopolysaccharides linked to the macrophage TLR4 promote IL-10 production by the macrophage through a mitogen-activated protein kinase (MAPK) signaling pathway (extracellular signal-regulated kinase [ERK], MAPK p38) and transcription factors such as CREB, NF-κB, and homodimeric p50.^39^ Consequently, we can suppose that the source of IL-10 could be macrophages.

In addition, we found that both infected and uninfected HbSS subjects had significantly higher IL-10 plasma levels than uninfected HbAS and HbAA subjects. Macrophages have been found to be the major source of IL-10 in most inflammatory diseases,^37^ and they are among the major protagonists in the pathophysiology of SCD.^40^ Thus, increased IL-10 could be associated with a normal steady-state condition in patients with SCD.

After stimulation of PBMCs from healthy donors, bacterial antigens induced activation and proliferation of immune cells. Thus, an immune response was triggered, and the immunogenicity of the *E coli* strains was well verified.

The measurement of TNF-α and IL-6 after 24 hours of stimulation allows for the exploration of innate immune response. In the innate response, the synergistic gene expression of cytokines TNF-α, IL-6, and even IL-10 and IL-12p70 is due to the combined stimulation of the TLR ligands through the NF-κB, IFN regulatory factor, MAPK, phosphatidylinositol 3-kinases, and signal transducer and activator of transcription signaling pathways.^41^ In addition, gram-negative bacteria have been shown to induce high levels of TNF-α and IL-6 after 4 hours of stimulation of immune cells.^42^

Measuring IL-10 after 72 hours of stimulation allows for the exploration of the adaptive response. The *E coli* extracts used in this study induced significant production of IL-10. Whole *E coli* are effective inducers of IL-10 from human leukocytes.^43^ In addition, *E coli* strains responsible for asymptomatic bacteriuria can downregulate the immune response in host cells.^44^

The CD4^+^ T lymphocyte profile observed after 72 hours of stimulation showed that CD4^+^ T lymphocytes were not the source of IL-10 in this study. In the adaptive immune response, IL-10 is produced not only by CD4^+^ T lymphocytes but also by B lymphocytes and CD8^+^ T lymphocytes.^45^ For example, lipopolysaccharides induce an optimal production of IL-10 by B lymphocytes after prolonged stimulation through the signaling pathway requiring myeloid differentiation factor 88.^46^

After co-culturing bacterial strains with cells from healthy volunteer donors, *E coli* strains induced a high level of IL-10. The increased IL-10 production in HbSS subjects with asymptomatic UTI could be linked to different antigen expression by bacteria strains. Fully virulent uropathogenic *E coli* strains have been shown to cause symptomatic UTI with acute inflammation and a strong innate immune response and tissue damage.^47^

This study has limitations in that we were not able to investigate a large number of subjects, and because of the skepticism of patients with SCD, we were unable to recollect blood from them to stimulate their PBMCs with *E coli* antigens. Nevertheless, this study showed that HbSS subjects with asymptomatic UTI had elevated plasma levels of IFN-γ and elevated IL-10 levels.

Together, these findings may explain that IL-10 concentration may be responsible for the asymptomatic status of UTI in the patients studied.

## CONCLUSION

Our study demonstrated that HbSS subjects with UTIs had increased plasma levels of IFN-γ associated with high levels of IL-10, but T helper cells were not the main source of those cytokines. These findings could indicate that the IL-10 environment may be responsible for asymptomatic UTIs in patients with SCD. Further studies should investigate the source of plasma IL-10. Systematic cytobacteriologic examination of the urine of HbSS subjects would be of interest.

## Notes

*This article meets the Accreditation Council for Graduate Medical Education and the American Board of Medical Specialties Maintenance of Certification competencies for Patient Care and Medical Knowledge.*
